# Whole-genome sequencing and analysis of *Streptomyces* strains producing multiple antinematode drugs

**DOI:** 10.1186/s12864-022-08847-4

**Published:** 2022-08-23

**Authors:** Jeong Sang Yi, Jung Min Kim, Min-Kyoung Kang, Jong Hoon Kim, Hang Su Cho, Yeon Hee Ban, Myoung Chong Song, Kwang-Hee Son, Yeo Joon Yoon

**Affiliations:** 1 College of Pharmacy, Natural Products Research Institute, Seoul National University, 1 Gwanak-ro, Gwanak-gu, Seoul, 08826 Republic of Korea; 2 Industrial Biomaterial Research Center, Korea Research Institute of Bioscience and Biotechnology, 125 Gwahak-ro, Yuseong-gu, Daejeon, 34141 Republic of Korea

**Keywords:** *Streptomyces*, Secondary metabolite, Nematicidal, Spectinabilin, Undecylprodigiosin

## Abstract

**Background:**

Nematodes are parasitic animals that cause over 100 billion US dollars loss in agricultural business. The whole-genomes of two *Streptomyces* strains, *Streptomyces spectabilis* KCTC9218^T^ and *Streptomyces* sp. AN091965, were sequenced. Both strains produce spectinabilin, an antinematode drug. Its secondary metabolism was examined to aid the development of an efficient nematicidal drug-producing host strain.

**Results:**

The whole-genome sequences of *S. spectabilis* KCTC9218^T^ and *Streptomyces* sp. AN091965 were analyzed using PacBio and Illumina sequencing platforms, and assembled using hybrid methodology. The total contig lengths for KCTC9218^T^ and AN091965 were 9.97 Mb and 9.84 Mb, respectively. A total of 8,374 and 8,054 protein-coding genes, as well as 39 and 45 secondary metabolite biosynthetic gene clusters were identified in KCTC9218^T^ and AN091965, respectively. 18.4 ± 6.45 mg/L and 213.89 ± 21.30 mg/L of spectinabilin were produced by *S. spectabilis* KCTC9218^T^ and *Streptomyces* sp. AN091965, respectively. Pine wilt disease caused by nematode was successfully prevented by lower concentration of spectinabilin injection than that of abamectin recommended by its manufacturer. Production of multiple antinematode drugs, including spectinabilin, streptorubin B, and undecylprodigiosin was observed in both strains using high-resolution liquid chromatography mass spectrometry (LC–MS) analysis.

**Conclusions:**

Whole-genome sequencing of spectinabilin-producing strains, coupled with bioinformatics and mass spectrometry analyses, revealed the production of multiple nematicidal drugs in the KCTC9218^T^ and AN091965 strains. Especially, *Streptomyces* sp. AN091965 showed high production level of spectinabilin, and this study provides crucial information for the development of potential nematicidal drug producers.

**Supplementary Information:**

The online version contains supplementary material available at 10.1186/s12864-022-08847-4.

## Background

Nematodes are parasites that cause harm to humans, other animals, and plants [1]. Nematode infections in humans result in gastrointestinal diseases such as inflammatory bowel disease, and skin allergic reactions [2–4]. In addition, they cause various types of damages including stunting and yellow leaves in plants [5, 6]. The abundance of nematodes ranges from a dozen to over a thousand individual per 100 g of soil, depending on the geographic region. They are mostly found in the tundra, with a median of 2,695 nematodes per 100 g of dry soil [7]. This makes nematodes the most abundant animals on earth [8]. Nematode infections resulted in a reported loss of over 100 billion US dollars in 2018, [9] making them a major cause of financial strain in the agricultural sector. As a result, there have been more efforts to eliminate nematodes from the soil, with *Streptomyces* species as a promising source for the development of potent and environmentally-friendly nematicidal agents [10].

*Streptomyces* species are well known to produce various pharmaceutical products, including antibiotics and anticancer drugs [11, 12], as well as valuable agricultural chemicals [13]. Nematicides are no exception, and many have been discovered from members of the *Streptomyces* genus. Avermectin, isolated from *Streptomyces avermitilis*, has been one of the most widely used nematicides since its discovery in 1978 in Japan. In 2020, the annual global sales of avermectin reached $850 million [14]. The avermectin class of molecules includes ivermectin, moxidectin, selamectin, abamectin, and nemadectin [15]. This nematicide interferes with glutamate-gated chloride channels and GABA receptors, resulting in the restriction of movement and reproduction, which leads to nematode death [16]. Spectinabilin (also known as neoaureothin), another potent nematicide, was discovered in *Streptomyces spectabilis* in 1976 [17, 18] and is produced from chorismate, five molecules of methylmalonyl-CoA, and one malonyl-CoA by polyketide synthase (PKS) [19]. The detailed mode of action of spectinabilin against nematodes is yet to be determined, but it is one of the only two drugs that cause vacuolar death. Treatment of nematode-infected pine trees with spectinabilin increases the survival rate of the trees from 10 to 80% [20, 21]. Prodigiosin, a member of the prodiginine group of compounds produced by several *Streptomyces* species, is also one of the well-studied nematicide [22]. Biosynthesis of prodigiosin or its close derivative, undecylprodigiosin, (also known as RED due to its color) begins with proline and undergoes condensation with malonyl-CoA and serine, followed by hybridization with fatty acids [23, 24]. However, the mechanism of action of prodigiosin against nematodes also remains unknown.

Here, we present the genome sequences of two *Streptomyces* strains, *Streptomyces spectabilis* KCTC9218^T^ and *Streptomyces* sp. AN091965, which can produce antinematode agents. They were sequenced using both short-read Illumina and long-read PacBio technologies to obtain high-quality genomic sequences. Comparative genome analysis confirmed the presence of spectinabilin and the RED biosynthetic gene cluster (BGC). 18.4 ± 6.45 mg/L and 213.89 ± 21.30 mg/L of spectinabilin were produced by *S. spectabilis* KCTC9218^T^ and *Streptomyces* sp. AN091965, respectively. Especially for *Streptomyces* sp. AN091965, spectinabilin production level was tenfold higher than that from previous reports [25]. In addition, spectinabilin was more effective than abamectin in the prevention of pine wilt disease (PWD) in pine wood, *Pinus densiflora*, caused by nematode, *Bursaphelenchus xylophilus*. Further analysis of bacterial culture extracts revealed that the two *Streptomyces* strains actively produced multiple nematicidal drugs: spectinabilin, RED, and streptorubin B, another member of the prodiginine produced by RED BGC. Our study highlights the possibility of developing a highly potent nematicide-producing microbial strain for agricultural use.

## Results and discussion

### Strain isolation and characterization of *Streptomyces* sp. AN091965

In our previous study, we identified 29 *Streptomyces* strains that have nematicidal activities from 5000 actinomycete isolates. The mortality rate of these strains was above 80% against *B. xylophilus* [26]. They were isolated from forest soil samples collected from Chollipo Arboretum, Taean-gun, Chungcheongnam-do, Korea (36°47′56.6"N 126°08′58.3"E) in July 2009 (Fig. S1). Among the strains, *Streptomyces* sp. AN091965 was selected for further study because of its superior nematicidal properties [26]. In this study, the mortality rate of *Streptomyces* sp. AN091965 crude extract against *B. xylophilus* was quantitatively determined to be 98.1 ± 2.9%. Only the morphology of *Streptomyces* sp. AN091965 colonies was briefly reported in a previous study [26]; thus, more detailed morphological analyses, including physiological analysis, were carried out.

**Fig. 1:**
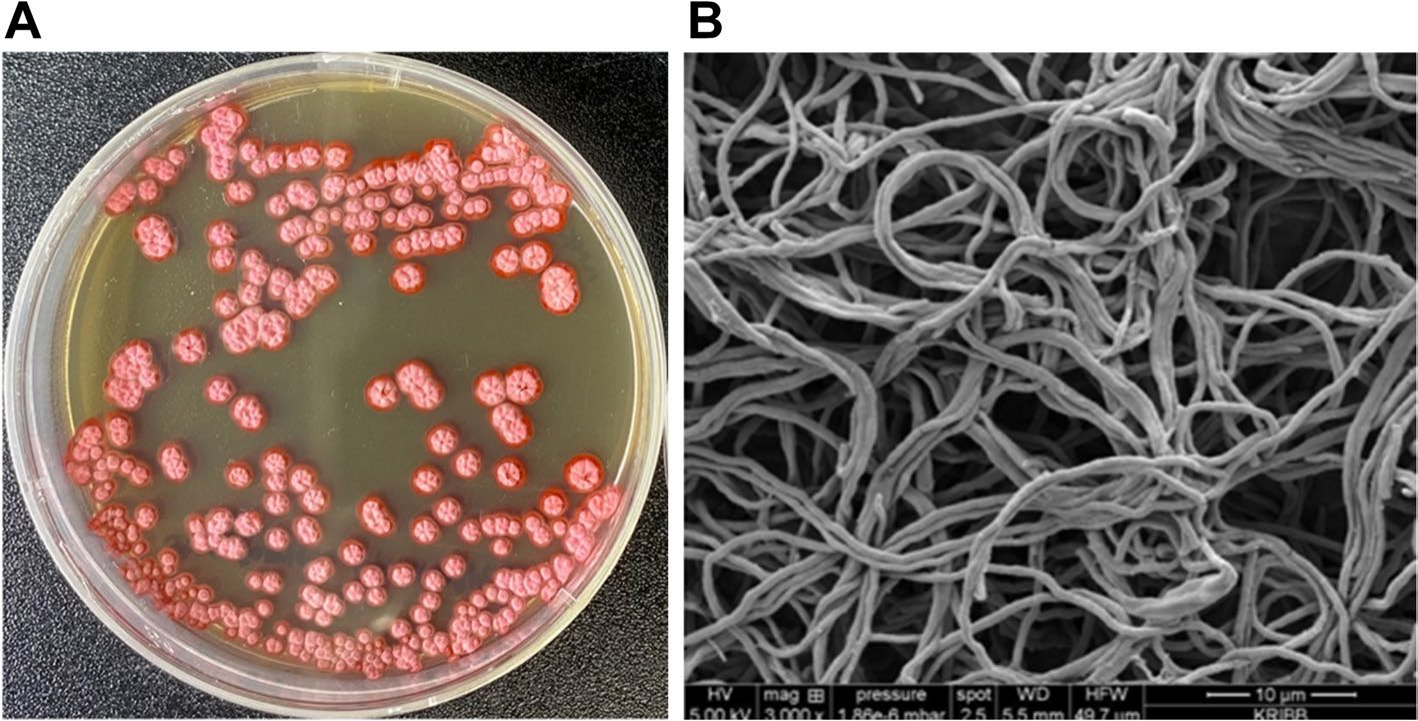
**A** Morphological characteristics and **B** aerial mycelia morphology under scanning electron microscope of *Streptomyces* sp. AN091965 grown on Bennett’s agar media

The AN091965 strain was found to grow on Bennett’s agar, Reasoner’s 2A (R2A) agar, and other International Streptomyces Project (ISP) media. The phenotypes of the strain are listed in Table 1. Strain AN091965 grew slowly on ISP 5 medium but grew moderately to rapidly on the other test media. Diffusible pigments were not produced in any of the media tested, except for the G.S.S. liquid culture media. Aerial mycelia were observed only on the Bennett’s agar medium. The substrate mycelia in all the tested media, except ISP 2, were red in color (Fig. 1A), and the development of spore-like bodies was not observed in the aerial mycelium in any of the media tested (Fig. 1B). Physiology of the *S. spectabilis* KCTC9218^T^ was very similar to that of AN091965 strain (Table S1; Fig. S2).

**Table 1:** Cultural characteristics of *Streptomyces* sp. AN091965 on different growth media

Strain	Medium	Cell growth	Colors of		
			**Aerial mycelia**	**Substrate mycelia**	**Soluble pigment**
AN091965	ISP 2	Moderate	-	Pale yellow	-
	ISP 3	Good	-	Red	-
	ISP 4	Moderate	-	Red	-
	ISP 5	Slow	-	Orange red	-
	R2A	Moderate	-	Orange red	-
	Bennett’s	Good	Light pink	Red	-

### Genomic feature and phylogenetic study

*S. spectabilis* KCTC9218^T^ is one of the best-known spectinabilin producers. It shared 99.86% 16S rRNA sequence similarity with *Streptomyces* sp. AN091965 in our previous study [26]. The high homology of 16S rRNA gene sequences between these two strains suggests that *Streptomyces* sp. AN091965 may also be a spectinabilin producer. Production of spectinabilin by *S. spectabilis* KCTC9218^T^ and a new *Streptomyces* sp. AN091965 was confirmed using high-resolution liquid chromatography mass spectrometry (LC–MS) analysis (Fig. S3).

**Fig. 2:**
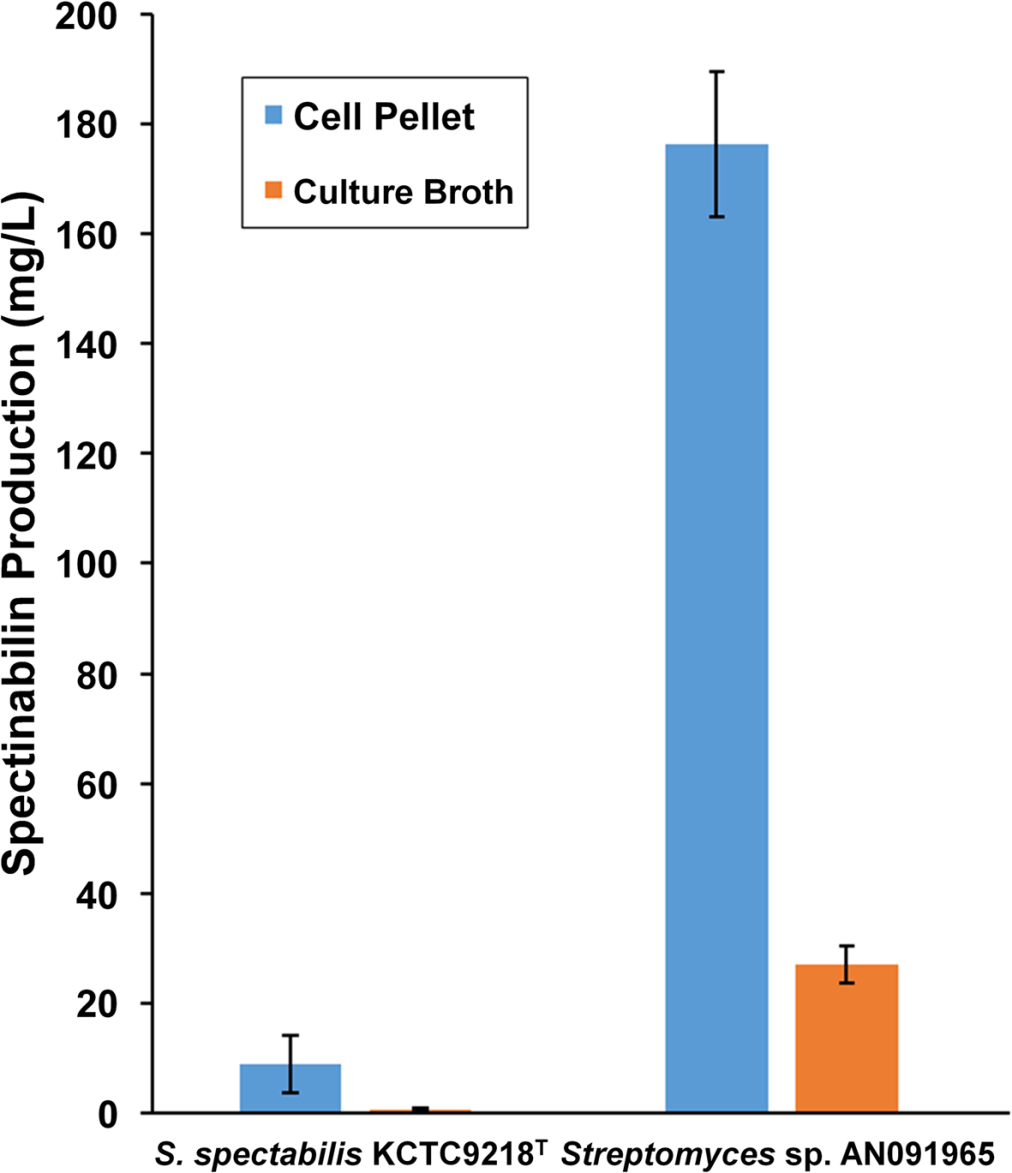
Quantification of spectinabilin from *Streptomyces* sp. AN091965 and *S. spectabilis* KCTC9218^T^

Spectinabilin was extracted from the cell pellet and culture broth separately, and a major amount of the compound (approximately 86% and 89% from KCTC9218^T^ and AN091965, respectively) was extracted from the cell pellets, indicating that it was not secreted efficiently from either strain (Fig. 2). 16.08 ± 5.06 mg/L and 185.57 ± 13.20 mg/L of spectinabilin were extracted from cell pellets, while 2.32 ± 1.39 mg/L and 28.32 ± 8.10 mg/L were extracted from culture broth of *S. spectabilis* KCTC9218^T^ and *Streptomyces* sp. AN091965, respectively. Production of spectinabilin from AN091965 was over tenfold higher than that from KCTC9218^T^ and *Streptomyces orinoci* ATCC23202^T^ [26], one of the strains identified earlier as a spectinabilin producer. In our previous study, AN091965 was identified to have the highest level of nematicidal activity among 29 nematicide producing strains we isolated [26]. Such significant differences in nematicidal activity of AN091965 may be explained by the extraordinary levels of spectinabilin production. Further experiments such as RNA-seq and metabolite profiling would be required to explain the differences between secondary metabolism of *S. spectabilis* KCTC9218^T^ and *Streptomyces* sp. AN091965, but AN091965 bears a potential for industrial scale production of spectinabilin by further engineering.

**Fig. 3:**
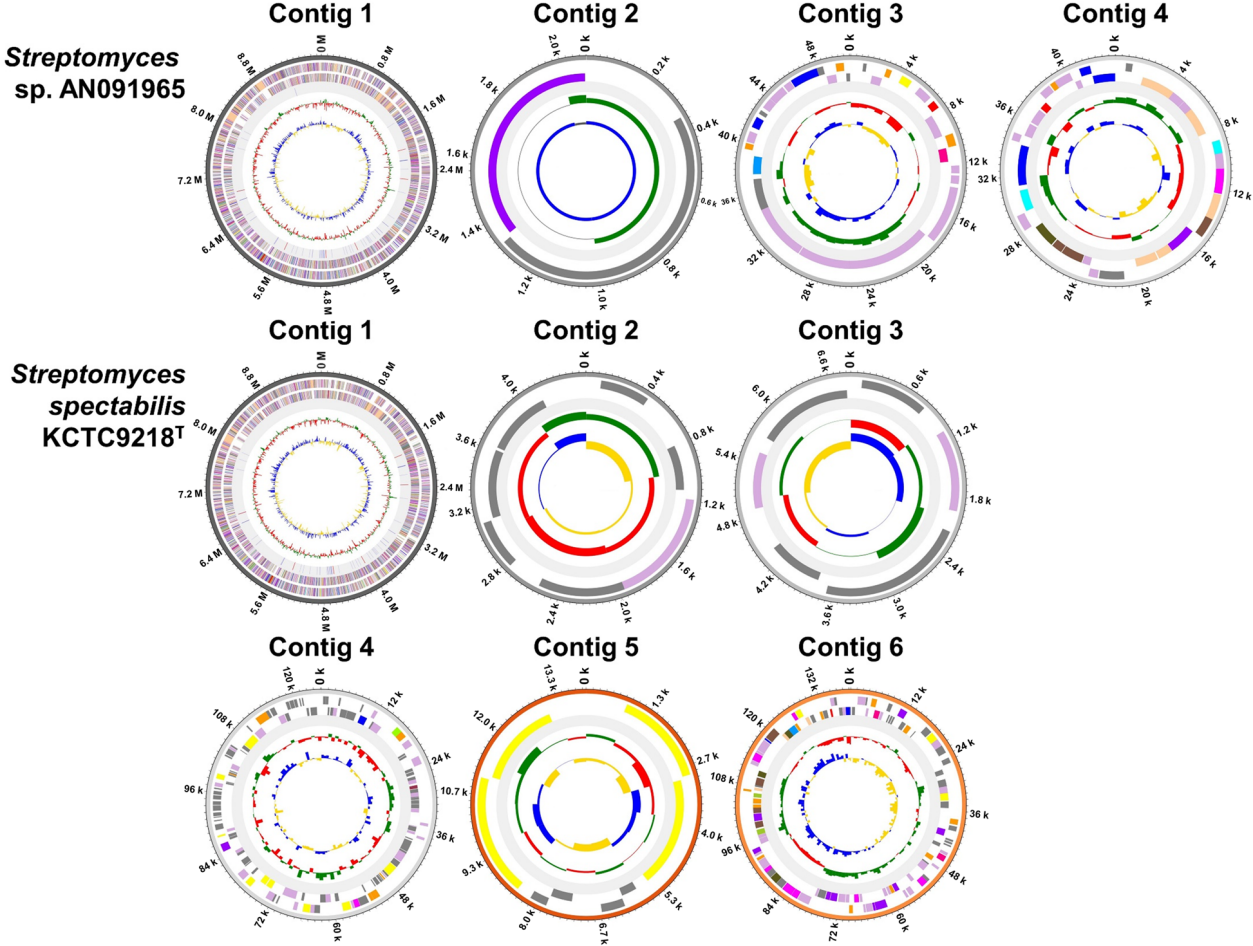
Circular maps of *Streptomyces* sp. AN091965 (top) and *S. spectabilis* KCTC9218^T^ (bottom) contigs. Description of each circle is presented from the outmost. (1) Size of contigs presented in million base pairs (M) or killo base pairs (k). (2) Predicted coding sequences of the forward strands. (3) Predicted coding sequences of the reverse strands. (4) Positions of rRNA and tRNA. (5) GC Skew, and (6) GC ratio. Contig breaks occur at 0 M or 0 k markers

To better understand the genomic features of the two strains, high-quality draft genome sequences from *S. spectabilis* KCTC9218^T^ and *Streptomyces* sp. AN091965, consisting of six and four contigs, respectively, were obtained using PacBio and Illumina sequencing technologies, with a hybrid assembly of the two (Fig. 3). The total contig length was 9,967,045 bp for KCTC9218^T^, with an average G + C content of 72.4%, and 9,837,143 bp for AN091965, with an average G + C content of 72.7%. In total, 8,374 protein-coding genes (CDS), 18 rRNA coding genes, and 64 tRNA coding genes were present in KCTC9218^T^. A total of 8,054 CDS, 18 rRNA, and 67 tRNA genes were identified in AN091965. Detailed genomic data on KCTC9218^T^ and AN091965 is provided in Table 2.

**Table 2:** Genomic features of *S. spectabilis* KCTC9218^T^ and *Streptomyces* sp. AN091965

	**KCTC9218**^**T**^	**AN091965**
Genome size (bp)	9,677,765	9,837,143
GC content (%)	72.4	72.7
Contigs	6	4
rRNA genes	18	18
tRNA genes	64	67
Protein-coding genes	8,374	8,054
Mean of gene length (bp)	1,051	1,077
Secondary metabolite gene clusters	45	39
Genes assigned to COG	7244	7179
Close relative by the initial 16S rRNA sequencing	*Streptomyces spectabilis* ATCC27465^T^ (96.93%)	*Streptomyces spectabilis* ATCC27465^T^ (96.38%)
Close relative by 16S rRNA gene sequences from whole genome sequencing	*Streptomyces spectabilis* ATCC27465^T^ (100%) (with KCTC9218^T^-orf01547)	*Streptomyces spectabilis* ATCC27465^T^ (99.74%) (with AN091965-orf01633)

**Fig. 4:**
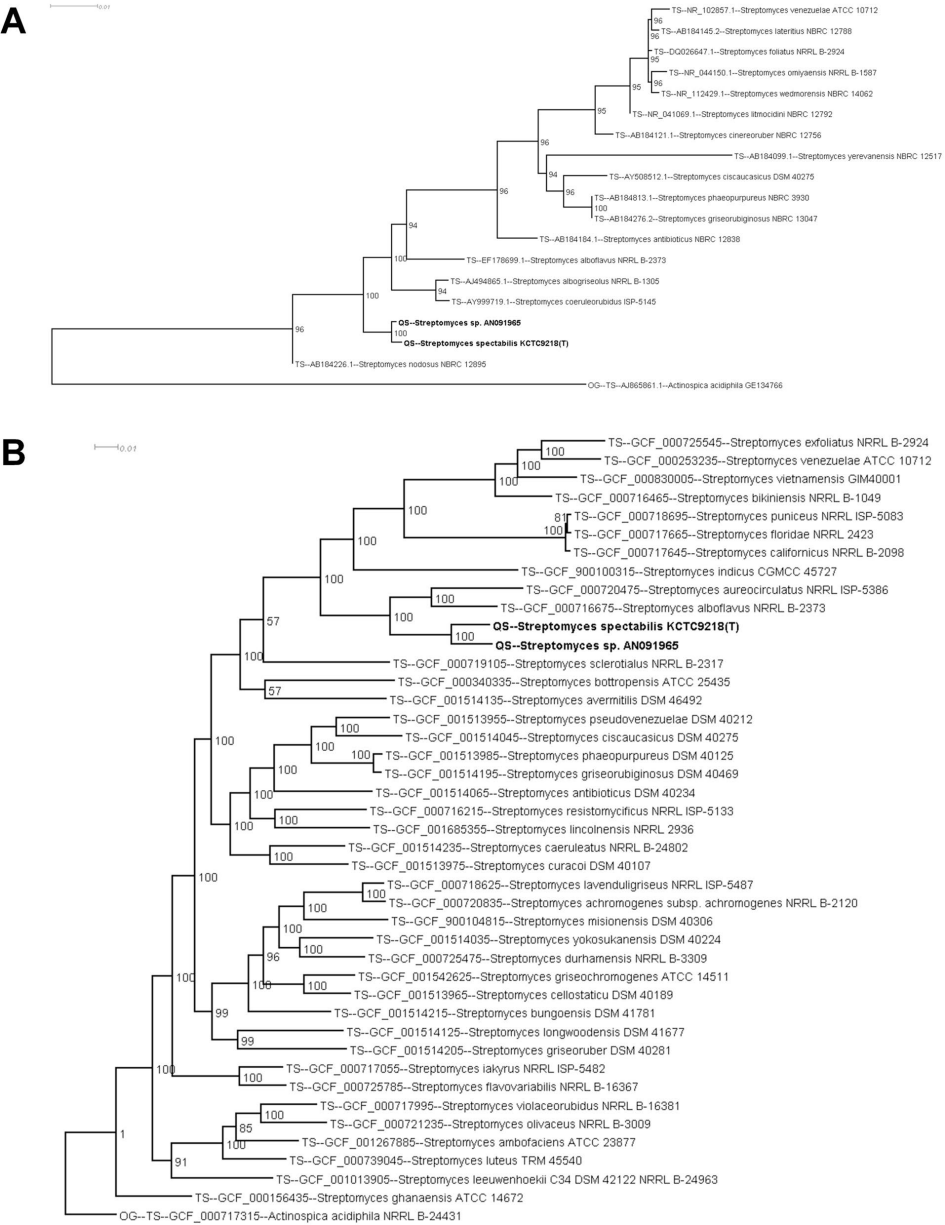
Phylogenetic tree of **A** the 16 s rRNA sequences of *S. spectabilis* KCTC9218^T^ and *Streptomyces* sp. AN091965 constructed by MEGAX, and **B** multi-locus species by autoMLST. TS represents type strain, QS represents query sequence, and OG represents out group. Accession numbers of the 16S rRNA gene or whole genome sequences are indicated between the type of strains and strain names. The two QS strains, highlighted by bold texts, are spectinabilin-producing *Streptomyces* strains. Bootstrap values are expressed as percentages

Phylogenetic analysis of *S. spectabilis* KCTC9218^T^ and *Streptomyces* sp. AN091965 was first conducted by MEGAX with 16S rRNA sequences (Fig. 4A). Besides *S. spectabilis* ATCC27465^T^ strain, close relative of the two strains were *Streptomyces coeruleorubidus* ISP-5145^ T^ (97.73% and 97.73% 16S rRNA gene sequence similarities for AN091965 and KCTC9218^T^, respectively) and *Streptomyces albogriseolus* NRRL B-1305^ T^ (98.29% and 98.22% for AN091965 and KCTC9218^T^, respectively). Next, the taxonomic positions of the two strains were analyzed using autoMLST software. A total of 43 strains, including two spectinabilin-producing *Streptomyces* species, 40 type *Streptomyces* strains, and one out-group strain, were analyzed (Fig. 4B). The autoMLST software provides high-quality genome sequence datasets from archives and determines the phylogeny of tested strains by Multi-Locus Sequence Analysis, also known as MLSA, comparing single-copy homologous genes shared among the type and out-group strains [27]. More detailed phylogeny of the analyzed strains can be constructed in this way. All the selected genes for MLST analysis are listed in Table S2. KCTC9218^T^ and AN091965 were identified as close relatives of *Streptomyces aureocirculatus* NRRL ISP-5386^ T^ and *Streptomyces alboflavus* NRRL B-2373^ T^.

### Analysis of genomic features

**Fig. 5:**
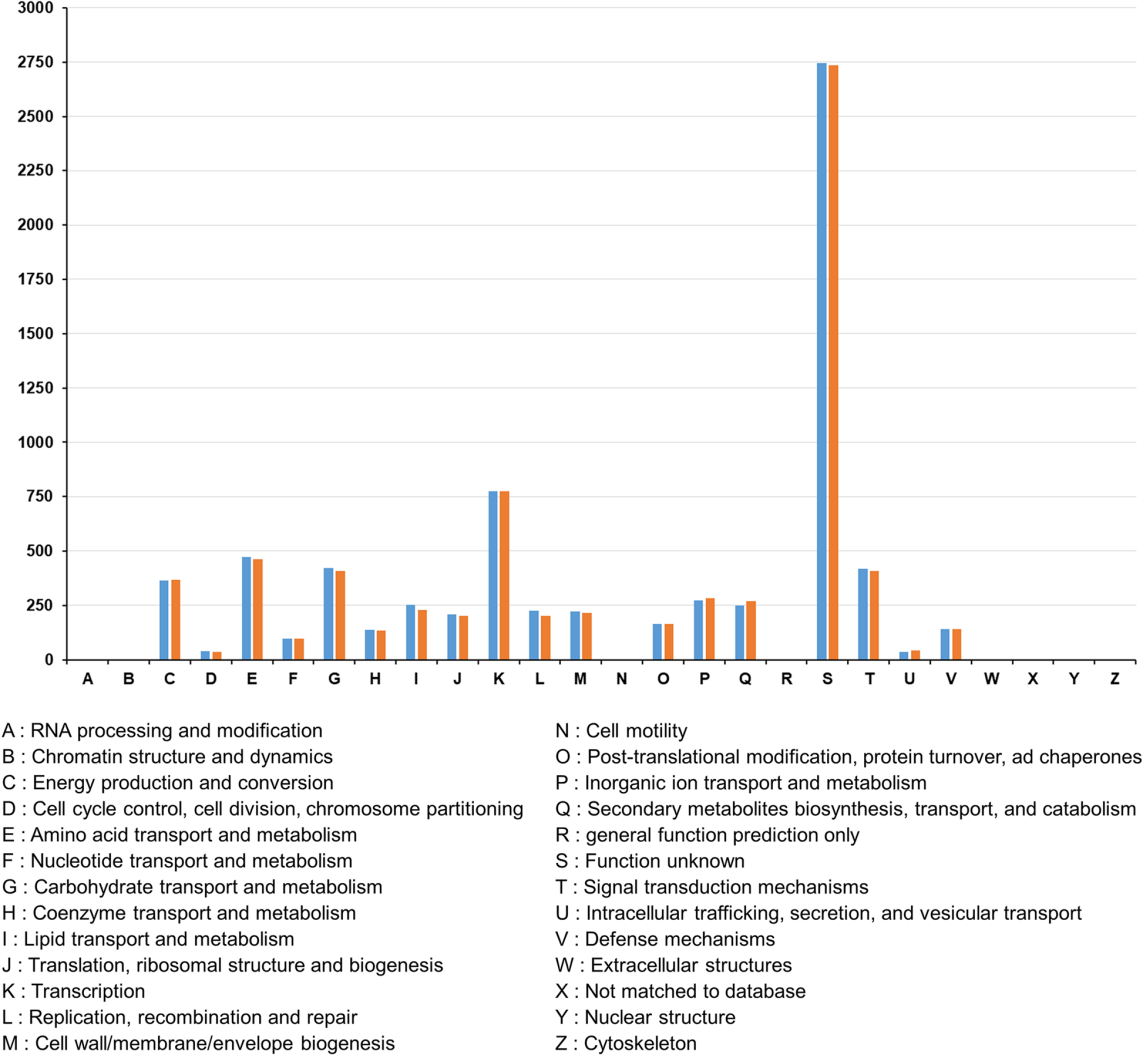
EggNog analysis of the two spectinabilin-producing strains

CDSs from KCTC9218^T^ and AN091965 were analyzed and assigned to Clusters of Orthologous Groups (COG) by EggNOG [28] (Fig. 5). A total of 7902 CDSs from KCTC9218^T^ and 7667 CDSs from AN091965 were assigned to COG. The largest proportion of all genes belonged to the S category. (Genes with unknown function): 2747 genes from KCTC9218^T^ and 2737 genes from AN091965 were assigned to category S, which accounted for 37.92% and 38.13% of the entire CDSs, respectively. Genes related to transcription were the second largest COG, with 10.67% for KCTC9218^T^ and 10.80% for AN091965. Genes coupled with amino acid transport and metabolism accounted for 6.52% for KCTC9218^T^ and 6.43% for AN091965, whereas carbohydrate transport and metabolism accounted for 5.83% in KCTC9218^T^ and 5.67% in AN091965. The distribution of the annotated genes was similar to that of various other *Streptomyces* strains [29], as transcription and primary metabolism, including carbohydrate and amino acid metabolism, are among the most essential features in living organisms. The distribution of all other genes among the COG categories was also similar between the two spectinabilin-producing *Streptomyces* strains.

**Fig. 6:**
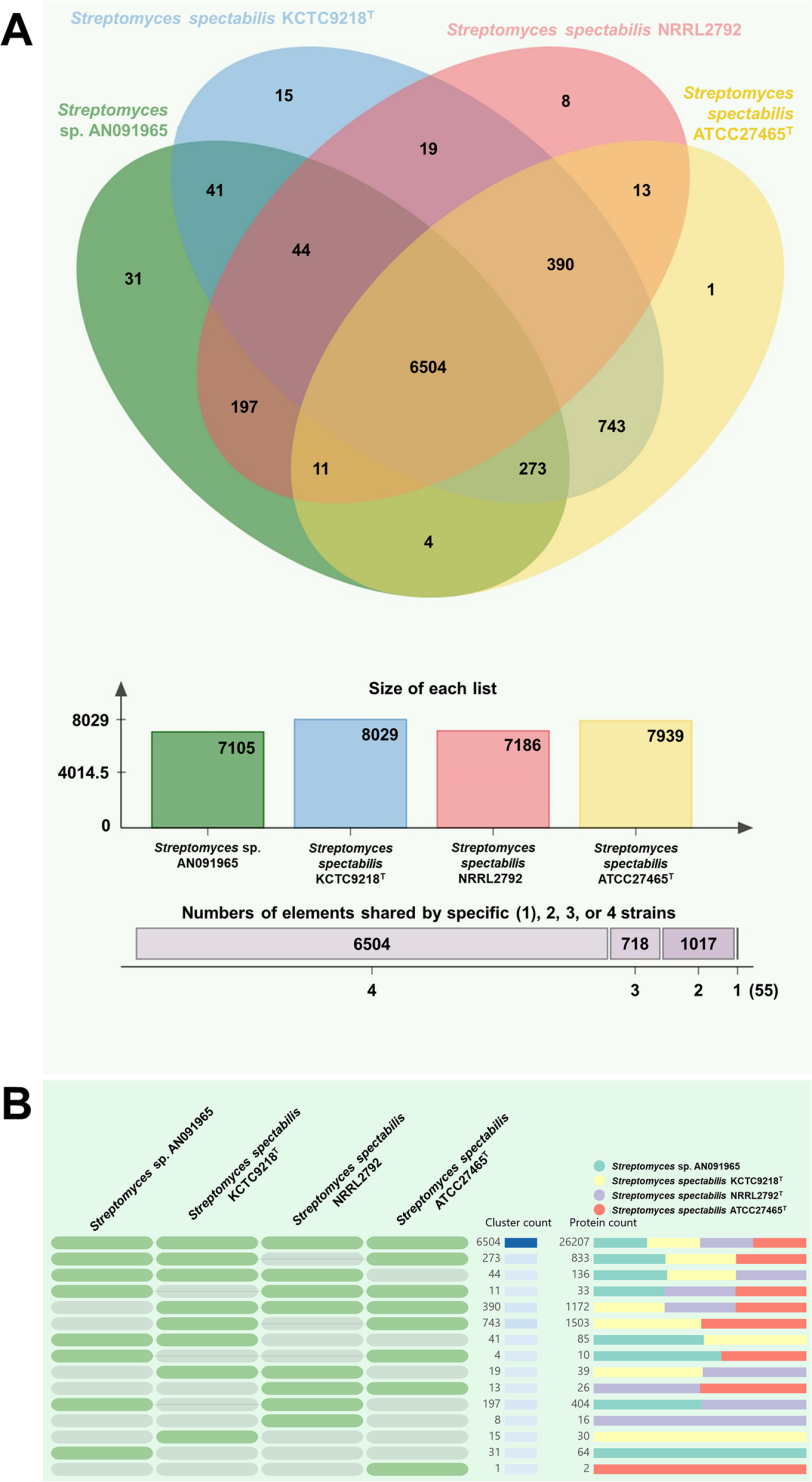
**A** Venn diagram of orthologous gene clusters present among *Streptomyces* sp. AN091965 and the other three *S. spectabilis* strains, and **B** table-like representation of the diagram created by OrthoVenn2. Cluster and protein count indicate numbers of categories in functions of orthologous genes, and the total numbers of the orthologous genes in that categories, respectively. Orthologous gene clusters present in each strain are indicated by bold green under the strain names

Phylogenetic analysis revealed that KCTC9218^T^ and AN091965 were closely related (Fig. 4B). The two strains also shared high similarities in genetic features and orthologous protein groups. The 16S rRNA sequences of KCTC9218^T^ and AN091965 acquired from whole genome sequencing data shared 100% and 99.74% identity with *Streptomyces spectabilis* ATCC27465^T^ (a well-known spectinabilin producer), respectively [30]. To examine the differences between the three spectinabilin-producing strains, especially for KCTC9218^T^ and ATCC27465^T^, having 100% identical 16S rRNA genes, the compositions of orthologous gene clusters were analyzed using OrthoVenn2 [31]. *Streptomyces spectabilis* NRRL2792 [32], another spectinabilin producing *Streptomyces* strain was included for the analysis (Fig. 6). A total of 8029 and 7105 orthologous gene clusters were found in KCTC9218^T^ and AN091965, respectively. In total, 6504 clusters were common among the four *Streptomyces* strains. A total of 41 clusters were unique in AN091965 and KCTC9218^T^ strains. 15 clusters from KCTC9218^T^ and 31 clusters from AN091965 were unique to themselves. One cluster for ATCC27465^T^, and 8 clusters from NRRL2792 were unique to themselves. Although the four spectinabilin-producing strains shared high genomic similarities, such as COG, there were distinctive differences in the distribution of predicted protein function of the orthologous clusters. Especially for KCTC9218^T^ with 100% identical 16S rRNA gene sequences compared to those of ATCC27465^T^, such differences in organization of orthologous gene clusters were remarkable. Previously, all eleven *Brevundimonas alba* strains isolated from freshwater community were verified to have 100% identical 16S rRNA gene sequences to each other, but determined to have great differences in their genomic contexts [33]. The KCTC9218^T^ and ATCC27465^T^ strains showed similar results. Therefore, whole genome sequencing of KCTC9218^T^ strain demonstrated the diversity of *S. spectabilis* community.

### Identification of secondary metabolite gene clusters using antiSMASH

**Fig. 7:**
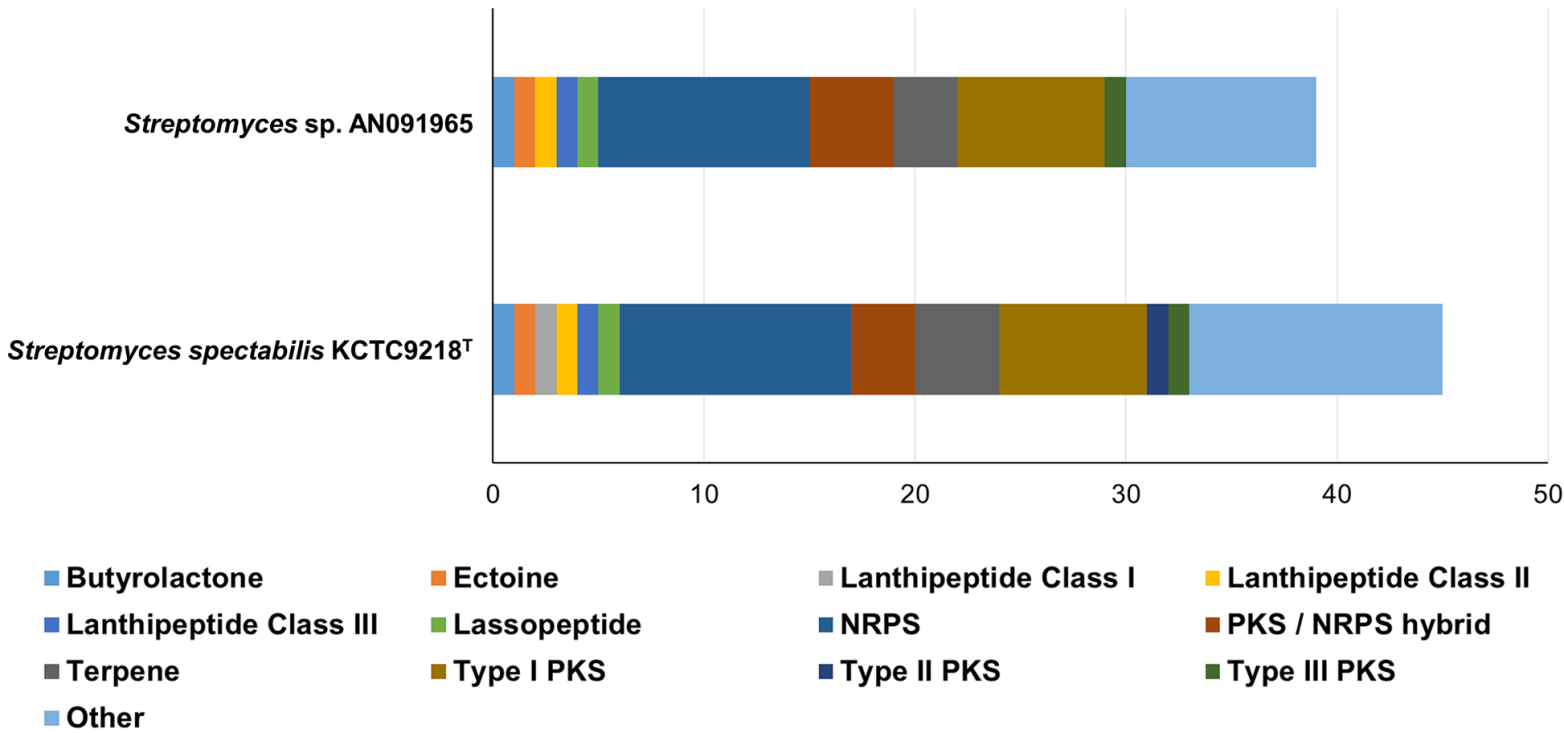
Secondary metabolite biosynthetic gene clusters predicted by antiSMASH. The other category includes incomplete gene clusters, single enzyme PKS, or single enzyme NRPS gene clusters. It also includes gene clusters only with non-essential or accessory genes such as post modification genes

The antiSMASH [34] analysis predicted 45 and 39 secondary metabolite BGCs in KCTC9218^T^ and AN091965, respectively, and we manually examined the predicted BGCs if they really contain the core biosynthetic genes (Fig. 7). This included the BGCs of nine PKSs, eleven nonribosomal peptide synthetases (NRPSs), and three PKS/NRPS hybrids from KCTC9218^T^. The BGCs of eight PKSs, ten NRPSs, and four PKS/NRPS hybrids were observed from AN091965. In both strains, NRPS was the most abundant secondary metabolite BGC. Type I PKS accounted for the most, seven in each strain, among the PKS BGCs. Three terpene BGCs, namely hopene, albaflavenone, and geosmin, were present in both KCTC9218^T^ and AN091965 (Table 3). Despite differences in orthologous protein clusters, antiSMASH predictions of secondary metabolite BGCs were very similar between KCTC9218^T^ and AN091965. There were 10 similar BGCs, which were expected to produce known compounds with identical predicted functions at high levels of similarity (> 80%) between the two strains. A notable difference was the position of lagmycin-like BGC on the largest contig. While the lagmycin BGC was located upstream of the ectoine BGC in contig 1 of KCTC9218^T^, it was located between actinospectacin and coelichelin BGCs in contig 1 of AN091965.

**Table 3:** List of known biosynthetic gene clusters identified by antiSMASH in the order of position on contig 1 of KCTC9218^T^ and AN091965

***S. spectabilis* KCTC9218**^T^			
Most probable cluster type	Most similar known cluster (% similarity)	Position on contig 1	
		From	To
Lassopeptide	Lagmycin (80%)	103,351	167,185
Ectoine	Ectoine (100%)	2,442,957	2,453,361
Terpene	Albaflavenone (100%)	6,162,482	6,181,854
Terpene	Geosmine (100%)	7,611,338	7,631,637
Terpene	Hopene (92%)	7,732,884	7,757,205
PKS / NRPS hybrid	Undecylprodigiosin (90%)	7,762,605	7,830,726
Type I PKS	Spectinabilin (90%)	7,845,810	7,919,250
Type I PKS	Streptovaricin (95%)	8,097,725	8,200,754
Aminocyclitol	Actinospectacin (100%)	8,470,375	8,574,169
NRPS	Coelichelin (100%)	9,117,449	9,167,358
***Streptomyces* sp. AN091965**			
Most probable cluster type	Most similar known cluster (% similarity)	Position on contig 1	
		From	To
Ectoine	Ectoine (100%)	2,664,967	2,675,371
Terpene	Albaflavenone (100%)	6,368,505	6,388,055
Terpene	Geosmine (100%)	7,746,404	7,766,638
Terpene	Hopene (92%)	7,871,449	7,895,767
PKS / NRPS hybrid	Undecylprodigiosin (86%)	7,902,098	7,971,207
Type I PKS	Spectinabilin (90%)	7,985,434	8,057,892
Type I PKS	Streptovaricin (95%)	8,261,520	8,355,976
Aminocyclitol	Actinospectacin (100%)	8,648,037	8,705,683
Lassopeptide	Lagmycin (80%)	9,339,601	9,362,230
NRPS	Coelichelin (100%)	9,378,474	9,427,437

### Comparison of spectinabilin BGCs

**Fig. 8:**
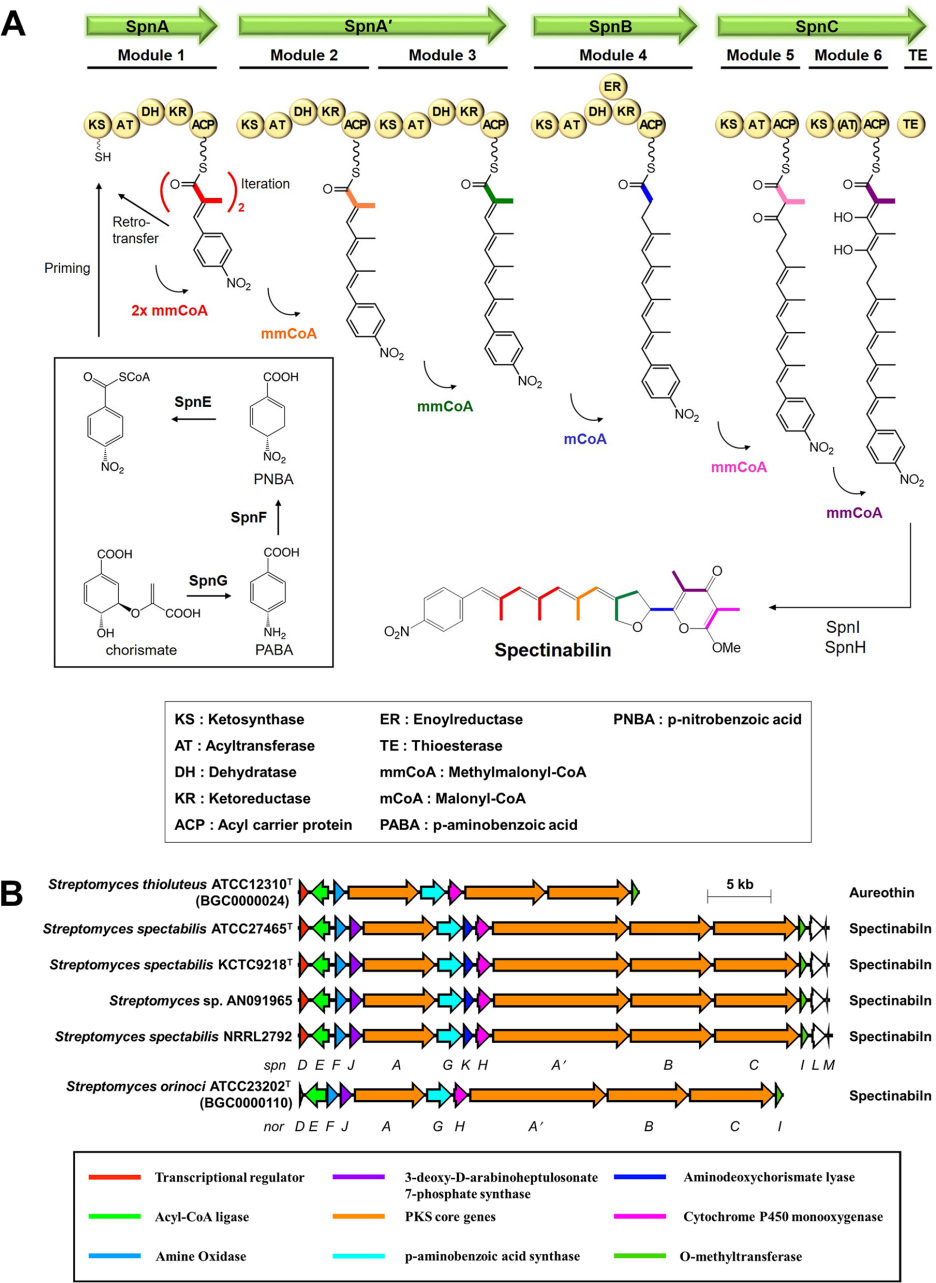
**A** Biosynthetic pathway of spectinabilin, and **B** comparison of spectinabilin BGCs from several spectinabilin-producing strains. Spectinabilin biosynthetic gene clusters are predicted by antiSMASH for comparison, or else MIBiG accession numbers are indicated under the strain names

Two different types of spectinabilin BGCs have been reported in *S. spectabilis* ATCC27465^T^ and *Streptomyces orinoci* ATCC23202^T^. Overall, spectinabilin is produced from seven modules of PKS, which are composed of four genes: *spnA*, *spnA′, spnB*, and *spnC* [19]. Spectinabilin biosynthesis is predicted to begin with a chorismate as a starting unit [25]. This is followed by polyketide chain elongation, with six methylmalonyl-CoA and one malonyl-CoA molecules as extender units (Fig. 8A). The major difference between the two spectinabilin BGCs is the function of a regulatory protein present in the cluster. SpnD (AfsR family transcriptional regulator) in *S. spectabilis* is a positive regulator of the BGC, whereas NorD (ArsR family transcriptional regulator) is a repressor in *S. orinoci* ATCC23202^T^ [35].

The spectinabilin BGCs from five producers and aureothin (a structurally and biosynthetically similar compound to spectinabilin) BGC from *S. thioluteus* were compared (Fig. 8B) to investigate the genetic structure of spectinabilin BGC and propose its biosynthetic mechanism in KCTC9218^T^ and AN091965 strains. The domain and modular organization of PKSs are identical in all spectinabilin-producers, which indicates that spectinabilin biosynthesis follows the same series of PKS-catalyzed reactions in all producers (Fig. 8A). The SpnD of KCTC9218^T^ and AN091965 shared 96.06% identity. When compared to SpnD of ATCC27465^T^, SpnD of KCTC9218^T^ and AN091965 shared 99.62% and 96.23% identity, respectively. However, no similarity with NorD of *S. orinoci* ATCC23202^T^ was observed. This suggests that SpnD of KCTC9218^T^ and AN091965 are positive regulators of spectinabilin BGC. In addition, the *spnK* gene was found in *S. spectabilis* ATCC27465^T^, KCTC9218^T^, and NRRL2792, *Streptomyces* sp. AN091965 [36], whereas it was absent in *S. orinoci* ATCC23202^T^ and *S. thioluteus* ATCC12310^T^. Although the specific function of *spnK* is not known, it is nonessential for spectinabilin production [36]. Finally, a putative spectinabilin membrane transporter, *spnM*, was identified in all strains.

Although there are high similarities in the spectinabilin BGC between KCTC9218^T^ and AN091965, it would require further studies to identify the cause of differences in spectinabilin production level of the two strains. Secondary metabolism is affected by many factors such as carbon and nitrogen metabolism, gene regulations, physical stress, and availability of nutrients in surroundings [37]. Gene regulation is a complex series of regulatory networks. Although a pathway-specific regulator governing spectinabilin production, SpnD, is identified, the complex upstream regulatory network affecting SpnD cannot be understood solely based on genome sequences. On the other hand, polyketides draw their precursors from carbon sources such as glucose and fatty acids, and the differences in carbon metabolism of KCTC9218^T^ and AN091965 should also be understood [38]. Further studies including metabolite profiling and RNA-seq in combination with genomic information may explain such differences in spectinabilin producing behavior.

### Nematicidal activity and toxicity test on nematode infected pine woods

**Fig. 9:**
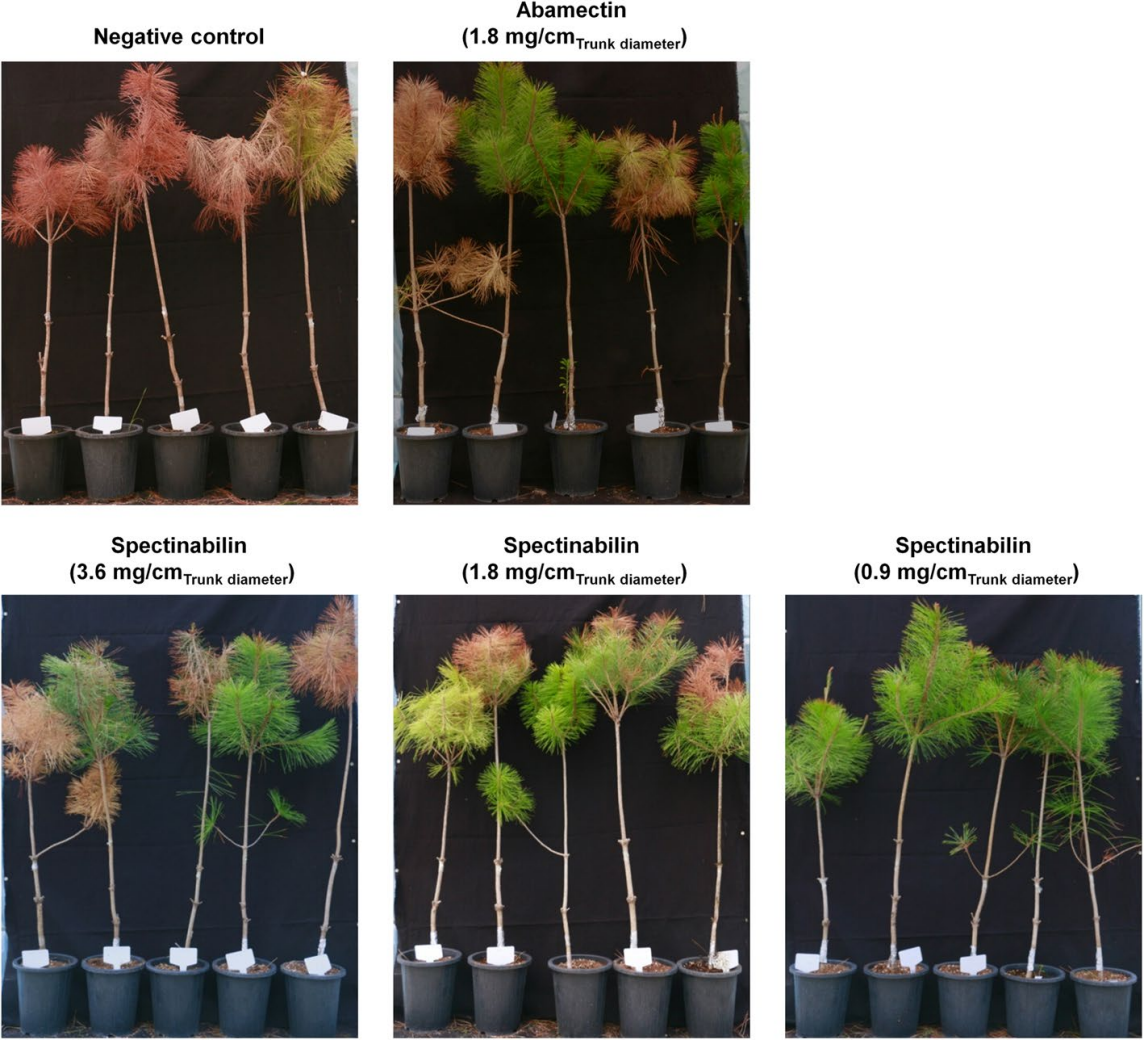
Evaluation of the efficacy of spectinabilin against *B. xylophilus*, pine wood nematode, under greenhouse conditions

*B. xylophilus* infection in trunks and branches of pine woods, causing PWD, is possibly due to phytotoxins released by the nematode [39]. To evaluate the nematicidal activities of spectinabilin, spectinabilin purified from *Streptomyces* sp. AN091965 (Fig. S4) was treated to pine wood, *Pinus densiflora*, before *B. xylophilus* infection (Fig. 9). Successful nematode infection in pine was visualized as the presence of needle discoloration, and these visual symptoms allowed us to evaluate the effect of spectinabilin on survival of the trees. 100% of nematicide untreated pine woods died after 60 days of *B. xylophilus* infection.

Spectinabilin at the concentration of 0.9 mg, 1.8 mg, and 3.6 mg per cm of pine wood trunk diameter was injected into trunks of pine woods before the *B. xylophilus* infection. The test was performed at quintuplicate, and commercially available nematicide, abamectin, was treated at 1.8 mg per cm of pine wood trunk diameter, a recommended concentration by its manufacturer, as a positive control. Supplement of spectinabilin or abamectin at the same concentration resulted in the prevention of nematode infection for three out of the five tested pine woods. Toxicity of spectinabilin to the pine woods was observed with increasing concentration, up to 3.6 mg/cm_Trunk diameter_. Spectinabilin treatment at 0.9 mg/cm_Trunk diameter_ successfully inhibited the development of PWD in all pine trees that are artificially infected with *B. xylophilus* from the quintuplicate test, implying that lower PWD prevention rate at 1.8 mg/cm_Trunk diameter_ spectinabilin treatment may have been due to the toxicity to the tree.

Alhough the rates of PWD outbreak was suppressed in pine woods by abamectin and spectinabilin treatments, some portion of *B. xylophilus* still survived from the nematicides. Thus, density of the nematode was measured. Number of *B. xylophilus* in the nematicide untreated pine wood increased from approximately 2000 to 3572 ± 322 per gram of trunk tissue. Abamectin and spectinabilin injection at the concentration of 1.8 mg/cm_Tree diameter_ decreased *B. xylophilus* density from approximately 2000 to 453 ± 51 and 211 ± 53 per gram of trunk tissue, respectively. This result indicated that spectinabilin was more effective than abamectin in the treatment of *B. xylophilus* infection. At the concentration of 0.9 mg/cm_Trunk diameter_ of spectinabilin injection, number of nematodes decreased from approximately 2000 to 298 ± 93 per gram of trunk tissue.

### Analysis of secondary metabolite production

The presence of secondary metabolite BGCs does not indicate that the gene clusters would actively produce the corresponding compounds. Secondary metabolism is affected by many factors, including environmental conditions and the type of media used for bacterial cultures [40, 41]. Cryptic BGCs are functional only under defined conditions [42]. Therefore, KCTC9218^T^ and AN091965 strains were cultured under standard laboratory conditions [43] using G.S.S. media to investigate whether they produce other nematicidal compounds other than spectinabilin. We reasoned that the red colors of KCTC9218^T^ and AN091965 cells, and antiSMASH prediction of the presence of undecylprodigiosin BGC suggested the two strains are possible producers of prodigiosin-type secondary metabolites in these strains. Thus, LC–MS analysis of crude extracts from the two strains focused on a search for prodigiosin or its derivatives.

LC–MS analysis of extracts from the cell pellets and supernatants of KCTC9218^T^ and AN091965 cultures showed three major peaks. The major product was spectinabilin and the other two were streptorubin B and RED. Despite having complete BGCs of several known NRPS and PKS metabolites (Table 3), only three were detected under the culture conditions used in this study. Generally, RED is not secreted from cells and is often extracted from cell pellets for quantification [44]. Similarly, it was not detected in the supernatants of KCTC9218^T^ and AN091965 cells. However, secretion of streptorubin B was detected in both strains (Fig. S5). Streptorubin B and RED are known for their nematicidal activities [22]. All three bioactive compounds produced by KCTC9218^T^ and AN091965 exhibited antinematode activity, suggesting that these two strains have great potential as sources of antinematode drugs.

## Conclusion

In this study, the genomes of two antinematode agent-producing strains, *S. spectabilis* KCTC9218^T^ and *Streptomyces* sp. AN091965, were sequenced and annotated. These strains were found to possess an additional secondary metabolite (RED) BGC, in addition to spectinabilin BGC, with antinematicide potential. Liquid cultures of the two strains and identification of bioactive molecules confirmed the active production of multiple nematicidal compounds: spectinabilin, streptorubin B, and RED. Efficacy test against nematode revealed that spectinabilin, at only half of the recommended concentration of abamectin, was sufficient for prevention of PWD. Especially, *Streptomyces* sp. AN091965 strain has a great potential as an inexpensive industrial nematicidal drug producer due to its ability to produce 200 mg/L of spectinabilin, which is tenfold higher than other spectinabilin producers reported.

## Methods

### Strains and culture conditions

*S. spectabilis* KCTC9218^T^ and *Streptomyces* sp. AN091965 strains were cultured in Bennett’s agar media at 28 ℃ before they were transferred to G.S.S. media (10 g of soluble starch, 20 g of glucose, 25 g of soybean meal, 1 g of beef extract, 4 g of yeast extract, 2 g of NaCl, 0.25 g of K_2_HPO_4_, and 2 g of CaCO_3_ in 1 L of distilled water) for antibiotic production. Cultures in ISP2 medium (Becton, Dickinson and Company, New Jersey, USA) were used to extract high molecular weight DNA for whole-genome sequencing. All the 50 mL liquid cultures were carried out at 28 ℃. They were shaken at 180 rpm in 50 mL volume using 250 mL baffled glass flasks. Bacterial cultures were harvested on day 2 for extraction of high molecular weight DNA for whole genome sequencing, and on day 7 for extraction and quantification of secondary metabolites.

### Extraction of secondary metabolites

Spectinabilin, RED, and streptorubin B were extracted separately from the mycelium and media broth. Mycelia were isolated through centrifugation. The pellets were suspended in a 10 × vol. of 80% methanol and sonicated for 20 min. The supernatant was collected through centrifugation, 7 min at 5000 × g and room temperature. The cell debris were further removed using paper filtration. The three compounds were extracted from the media broth using an equal volume of EtOAc. The EtOAc fraction was harvested, dried, and resolved in 15 mL methanol. The extracts from both mycelium and media broth were run through Sep-Pak Vac 3 cc silica solid-phase extraction column (Waters, Massachusetts, USA). The silica column was wet with 3 mL methanol, and pre-conditioned with 3 mL of 75% n-hexane in EtOAc. 20 μL of mycelia extract or 100 μL of media broth extract was loaded to the silica column. And then, the three compounds were eluted from the silica column using 3 mL of 75% n-hexane in EtOAc. The eluents were dried using SpeedVac, and reconstituted with 1 mL of acetone for nematicidal activity screening or MeCN for MS analysis.

### Nematode culture conditions

*B. xylophilus* was obtained from the National Institute of Forest Science (Seoul, Korea). It was separated from the xylem of PWD-infected trees using the Baermann funnel technique. The nematodes were maintained on *B. cinerea*-cultured Potato Dextrose Agar (PDA; Becton, Dickinson and Company, New Jersey, USA) at 25 ℃ for seven days. Cultured nematodes were harvested using sterilized water and subsequently counted.

### Determination of nematicidal activity of *Streptomyces* sp. AN091965 crude extract

The nematicidal activity of *Streptomyces* sp. AN091965 was determined, with purified spectinabilin from *S. spectabilis* KCTC9218^T^ as a control. 5 μL of 0.5 mg/mL spectinabilin or *Streptomyces* sp. AN091965 crude extract in acetone was added to 50 nematodes in 95 μL suspension in a 96-well plate. After 24 h, the nematodes were observed under a microscope to determine mortality. If the nematode body was motionless and straight, it was considered dead. Mortality was calculated as follows:

$$\mathrm{Mortality }\left(\mathrm{\%}\right)=\frac{\mathrm{Dead nematodes}}{\mathrm{Total nematodes}}\times 100$$

### Morphological characterization of *Streptomyces* sp. AN091965

To investigate its morphological characteristics, the AN091965 strain was streaked on Bennett’s agar medium. This was cultured at 28 ℃ for 10 days, after which spore form, size, and color of the mycelia of the metal-bearing strain were observed using a scanning electron microscope (FEI Quanta 250 FEG; FEI).

The culture characteristics of strain AN091965 were determined on ISP2, ISP3, ISP4, ISP5, Bennett’s agar, and R2A agar (Becton, Dickinson and Company, New Jersey, USA) media. After incubation at 28 °C for seven days, the morphological characteristics of the strain were observed under an optical microscope (Nikon Labophot-2).

### High molecular weight DNA extraction

High-molecular-weight genomic DNA was extracted from *S. spectabilis* KCTC9218^T^ and *Streptomyces* sp. AN091965 using the phenol/chloroform extraction method. Cell pellets were collected and washed twice with 10 mL of distilled water in 50 mL conical tubes. A 10 mL lysis buffer containing 3 mg/mL lysozyme (Sigma-Aldrich, Missouri, USA) and 100 μg/mL RNase (Sigma-Aldrich, Missouri, USA) in TE buffer (25 mM Tris–HCl, pH 8.0, 25 mM EDTA, and 0.3 M sucrose) was added. Cells were lysed in the conical tubes at 37 ℃ for 1 h. Proteinase K in distilled water was added to the lysate at a final concentration of 100 μg/mL, and the mixture was incubated at 55 ℃ for 2 h. 8 mL of phenol/chloroform (1:1) were added to the lysate and mixed by inversion for 6 min. The mixture was centrifuged for 15 min at 5000 × g and 4 ℃. The aqueous phase was transferred to a fresh tube, and 6 volume of ethanol was added. The mixture was stored at –20 ℃ for 12 h to enable DNA precipitation. DNA pellets were washed with 5 mL of ice-cold 70% ethanol twice, and the high molecular weight DNA was dissolved in 3 mL of distilled water.

### Whole-genome sequencing and assembly

The quality of the extracted DNA was measured using a NanoDrop ND-1000 (Thermo Scientific, Massachusetts, USA) and a Qubit fluorometer (Thermo Scientific) following the manufacturers’ protocol. The 16S rRNA gene was also sequenced to identify foreign gene contamination in the DNA samples. Whole-genome sequencing was performed by DNAlink (Seoul, Republic of Korea) using the Sequel (Pacific Biosciences, California, USA) and Novaseq6000 (Illumina, California, USA) systems. Whole-genome assembly was conducted by ChunLab (Seoul, Republic of Korea) using their Illumina-PacBio hybrid assembly method. Protein-coding sequences from the assembled strains were analyzed by ChunLab. Assembled genome sequences of *Streptomyces* sp. AN091965 and *S. spectabilis* KCTC9218^T^ have been submitted to NCBI GenBank, under BioProject ID PRJNA817371, and BioSample accessions SAMN26764618 and SAMN26764619, respectively. The genome sequences are available from the NCBI Nucleotide database by searching accessions JALDMY000000000 and JALDMZ000000000 for *Streptomyces* sp. AN091965 and *S. spectabilis* KCTC9218^T^, respectively.

### Phylogenetic analysis

A phylogenetic tree of the two spectinabilin-producing strains was produced through MEGAX (https://www.megasoftware.net/) [45] and autoMLST [27]. Phylogenetic tree was constructed from Clustal Omega aligned DNA sequences using the Maximum Likelihood method based on the general time reversible model. It was evaluated with bootstrap analysis of 1000. autoMLST analysis was performed using the fast alignment mode (MAFFT FFT-NS-2) under advanced settings with bootstrap analysis of 1000 replicates (http://automlst.ziemertlab.com/analyze). 50 type strains, reference genomes, and 3 outgroup strains were provided by the software. We reduced the number of reference genomes to 37 by removing repetitive strains. Concatenated alignment option was used to build the phylogenetic tree.

### Bioinformatic analysis

The coding sequences of *S. spectabilis* KCTC9218^T^ and *Streptomyces* sp. AN091965 were annotated using EggNOG v5.0 (http://eggnog5.embl.de/#/app/home) [28]. A minimum hit 2-value of 0.001, minimum hit bit score of 60, minimum percent identity of 40, minimum percent of query coverage of 20, minimum percent of subject coverage of 20, auto tax scope, and non-electronic GO evidence settings were used for the analysis. Orthologous gene clusters were analyzed through OrthoVenn2 (https://orthovenn2.bioinfotoolkits.net/home) [31], using default settings, 1e-2 E-value, and a 1.5 inflation value. Genomic data of *S. avermitilis* and *S. coelicolor* were provided by OrthoVenn2, whereas that of *S. spectabilis* ATCC27465^T^ was acquired from NCBI. Further, antiSMASH analysis was used to identify putative secondary metabolite BGCs (https://antismash.secondarymetabolites.org/#!/start) [34].

### LC–MS analysis of nematicidal metabolite extracts

Spectinabilin, RED, and streptorubin B were identified and quantified using an XEVO® G2S Q-ToF mass spectrometer coupled with an Acquity I-Class system (Waters, Massachusetts, USA). 2 μL of sample were injected, and UPLC was operated at a flow rate of 0.4 mL/min with solvent A, 0.1% formic acid (FA), and solvent B, 80% MeCN with 0.1% FA. Solvent gradient was applied, starting from 40% A to reach 5% A by 10 min, a 2 min hold at 5% A, and returning to 40% A in 0.1 min with an additional 0.9 min hold. An Xselect™ CSH™ C18 (2.1 × 100 mm, 2.5 μm) column (Waters, Massachusetts, USA) was maintained at 40 ℃. The MS system was operated in the ESI mode with positive ionization. The analyzer was operated with an extended dynamic range at a 60,000 resolution. Mass correction was performed using leucine enkephalin (400 pg/μL, 50% MeCN with 0.1% FA) as a lockspray infused at a rate of 5 μL/min. Authentic spectinabilin was provided by Korea Research Institute of Bioscience and Biotechnology for quantification.

### Purification of spectinabilin

Spectinabilin was purified from the crude extract before the suppression test. Silica open column was made by suspending 400 mL volume of silica with 500 mL 50% n-hexane in ethyl acetate, and the silica emulsion was tightly packed in a vertically setup, cylinder type separation funnel. The silica open column was washed with 1.5 L of n-hexane–EtOAc (10:1 volume by volume). Compounds were eluted from the silica open column by n-hexane–EtOAc gradient system (10:1, 8:1, 6:1, 5:1, 4:1, 3:1, 2:1, and 1:1, each 1 L), and finally by 2 L of 100% MeOH. Each fraction was dried under rotary vacuum evaporator, and dissolved in 15 mL of MeCN for LC–MS analysis. Conditions for LC–MS were the same as that for the screening of spectinabilin production in the crude extract. A majority of spectinabilin, at high purity, was detected in 100% MeOH fraction (Fig. S4), and spectinabilin was collected from HPLC fractions.

### Suppression of PWD under greenhouse conditions

Pot experiments were performed following the procedure from our previous study [46] with some modifications. One month before the experiment, five-year-old *P. densiflora* trees were transplanted and stored in a greenhouse with temperature maintained around 22 ℃ to 25 ℃ at the Korea Research Institute of Bioscience and Biotechnology (Daejeon, Korea). Pine wood growing pots were watered every other day. The height and basal diameter of each tree used in the pot experiment were recorded on the day of nematicide injection. Chemical solutions of spectinabilin were prepared using DPPT as a solvent (20% DMSO, 20% propylene glycol monomethyl ether, 50% propylene glycol, and 10% Tween 20) at the final concentration of 0.9 and 1.8 mg per 100 μL of DPPT. 18 mg/mL of abamectin was prepared with DPPT solution as a positive control. DPPT solution without nematicides was treated as a negative control. A hole (5 mm depth × 3 mm diameter) was drilled on trunks of pine trees about 5 cm above the soil at an angle of 45°, and prepared nematicide solutions were injected into the holes. The holes were covered with parafilm to prevent dryness.

Another holes were drilled on the trunk of pine trees approximately 20 cm above the soil at an angle of 45°. A 0.1 mL aliquot of the nematode suspension (containing about 2,000 nematodes, mixture of adults and juveniles) was pipetted into the holes, and the holes were also covered with parafilm. Average size of the subject pine trees was 120 cm in height, and 1 cm in basal trunk diameter. The pot experiments for spectinabilin efficacy tests were conducted in April 2022. Five replicates of triplicate experiments were conducted for each concentration of 0.9, 1.8, and 3.6 mg per cm of trunk diameter.

### Nematode extraction from pine woods and counting for survival test

After the pot experiment, pine tissue samples were collected to quantify nematodes that survived from abamectin and spectinabilin treatments. 2 g of trunk tissue was weighed and transferred onto a gauze in a 50 mL conical tube, filled with 30 mL of distilled water. After soaking the tissue in water for 24 h, it was carefully removed with the gauze from the tube. Nematodes released from trunk tissues were allowed to settle down onto the bottom of the conical tube for 12 h. Water was removed carefully from the top, reducing down to the final volume of 10 mL. 100 μL suspension of nematodes from the 10 mL were spotted onto glass slide, and the nematodes were counted under a microscope. The nematode population was calculated as the number of nematodes per gram of tree trunk.

## Supplementary Information

**Additional file 1: Table S1.** Cultural characteristics of *Streptomyces spectabilis* KCTC9218^T^ on different growth media. **Table S2.** List of genes used for autoMLST analysis. **Fig. S1.** Location of the sampling sites in South Korea. Red marks indicate areas where soil samples were obtained. **Fig. S2.** Morphological characteristics of *Streptomyces spectabilis* KCTC9218^T^ grown on Bennett’s agar media. **Fig. S3.** LC-MS analysis of (A) spectinabilin extracted from the cell pellets and media broth of *S. spectabilis* KCTC9218^T^ and *Streptomyces* sp. AN091965 cultures and (B) their MS/MS fragmentation analysis. **Fig. S4.** LC-MS chromatogram of metabolite fractions collected during purification of spectinabilin from the crude extract of *Streptomyces* sp. AN091965 culture broth and cell pellets. Spectinabilin was eluted from silica column at 100 % methanol. **Fig. S5.** LC-MS spectrum of spectinabilin (yellow), undecylprodigiosin (orange), and streptorubin B (green) from *S. spectabilis* KCTC9218^T^ and *Streptomyces* sp. AN091965 cultures.

## Data Availability

Assembled genome sequences of *Streptomyces* sp. AN091965 and *S. spectabilis* KCTC9218^T^ have been submitted to NCBI GenBank, under BioProject ID PRJNA817371, and BioSample accessions SAMN26764618 and SAMN26764619 respectively. The genome sequences are available from the NCBI Nucleotide database by searching accessions JALDMY000000000 and JALDMZ000000000 for *Streptomyces* sp. AN091965 and *S. spectabilis* KCTC9218^T^ respectively.

## Abbreviations

CDS: Protein-coding genes
COG: Clusters of orthologous groups
BGC: Biosynthetic gene cluster
PKS: Polyketide synthase
NRPS: Nonribosomal peptide synthetase
RED: Undecylprodigiosin
PWD: Pine wilt disease
