# Supplementary material for: Whole-genome sequencing and analysis of Streptomyces strains producing multiple antinematode drugs
Source: BMC Genomics. 2022 Aug 23;23:610. doi: 10.1186/s12864-022-08847-4 (PMC9396898; doi:10.1186/s12864-022-08847-4)
Supplement: Supplementary file 1 — Additional file 1: Table S1. Cultural characteristics of Streptomyces spectabilis KCTC9218T on different growth media. Table S2. List of genes used for autoMLST analysis. Fig. S1. Location of the sampling sites in South Korea. Red marks indicate areas where soil samples were obtained. Fig. S2. Morphological characteristics of Streptomyces spectabilis KCTC9218T grown on Bennett’s agar media. Fig. S3. LC-MS analysis of (A) spectinabilin extracted from the cell pellets and media broth of S. spectabilis KCTC9218T and Streptomyces sp. AN091965 cultures and (B) their MS/MS fragmentation analysis. Fig. S4. LC-MS chromatogram of metabolite fractions collected during purification of spectinabilin from the crude extract of Streptomyces sp. AN091965 culture broth and cell pellets. Spectinabilin was eluted from silica column at 100 % methanol. Fig. S5. LC-MS spectrum of spectinabilin (yellow), undecylprodigiosin (orange), and streptorubin B (green) from S. spectabilis KCTC9218T and Streptomyces sp. AN091965 cultures. [file 12864_2022_8847_MOESM1_ESM.docx]

***Additional file 1***

**Table S1** Cultural characteristics of *Streptomyces spectabilis* KCTC9218^T^ on different growth media

| **Strain** | **Medium** | **Cell growth** | **Colors of** | | |
| --- | --- | --- | --- | --- | --- |
|  |  |  | **Aerial mycelia** | **Substrate mycelia** | **Soluble pigment** |
| KCTC9218^T^ | ISP 2 | Moderate |  | Pale yellow |  |
|  | ISP 3 | Good |  | Orange red |  |
|  | ISP 4 | Good |  | Orange red |  |
|  | ISP 5 | Moderate |  | Orange red |  |
|  | R2A | Good |  | Orange red |  |
|  | Bennett’s | Good | Orange | Orange red |  |

**Table S2** List of genes used for autoMLST analysis

| **Gene** | **Function** |
| --- | --- |
| *gatB* | aspartyl/glutamyl-tRNA(Asn/Gln) amidotransferase, B subunit |
| *prfA* | peptide chain release factor 1 |
| *rpsS*_bact | ribosomal protein uS19 |
| IMP_synth_hisH | imidazole glycerol phosphate synthase, glutamine amidotransferase subunit |
| *hutI* | imidazolonepropionase |
| L18_bact | ribosomal protein uL18 |
| *rsmG*_gidB | 16S rRNA (guanine(527)-N(7))-methyltransferase RsmG |
| *sufC* | FeS assembly ATPase SufC |
| *ftsE* | cell division ATP-binding protein FtsE |
| FGAM_synth_I | phosphoribosylformylglycinamidine synthase I |
| T6A_YjeE | tRNA threonylcarbamoyl adenosine modification protein YjeE |
| *argR*_whole | arginine repressor |
| FruBisAldo_II_A | fructose-bisphosphate aldolase, class II |
| his_9_HisN | histidinol-phosphatase |
| L11_bact | ribosomal protein uL11 |
| *recQ* | ATP-dependent DNA helicase RecQ |
| *nth* | endonuclease III |
| *fpg* | DNA-formamidopyrimidine glycosylase |
| *lon* | endopeptidase La |
| *truB* | tRNA pseudouridine(55) synthase |
| succ_dehyd_cytB | succinate dehydrogenase, cytochrome b556 subunit |
| poly_P_kin | polyphosphate kinase 1 |
| *alaDH* | alanine dehydrogenase |
| *rplA*_bact | ribosomal protein uL1 |
| *pyrF*_sub2 | orotidine 5'-phosphate decarboxylase |
| TIGR00482 | nicotinate (nicotinamide) nucleotide adenylyltransferase |
| FGAM_synth_II | phosphoribosylformylglycinamidine synthase II |
| *serB* | phosphoserine phosphatase SerB |
| SUF_scaf_2 | SUF system FeS assembly protein, NifU family |
| *trmU* | tRNA (5-methylaminomethyl-2-thiouridylate)-methyltransferase |
| rplM_bact | ribosomal protein uL13 |
| PLP_synth_Pdx2 | pyridoxal 5'-phosphate synthase, glutaminase subunit Pdx2 |
| *rpsG*_bact | ribosomal protein uS7 |
| *gyrB* | DNA gyrase, B subunit |
| *gyrA* | DNA gyrase, A subunit |
| *ilvA*_1Cterm | threonine ammonia-lyase |
| asp_carb_tr | aspartate carbamoyltransferase |
| *bioA* | adenosylmethionine-8-amino-7-oxononanoate transaminase |
| *rplS*_bact | ribosomal protein bL19 |
| *infC* | translation initiation factor IF-3 |
| TIGR00042 | non-canonical purine NTP pyrophosphatase, RdgB/HAM1 family |
| tRNA_CCA_actino | CCA tRNA nucleotidyltransferase |
| RNasePH | ribonuclease PH |
| *panK*_bact | pantothenate kinase |
| *ftsZ* | cell division protein FtsZ |
| *ftsY* | signal recognition particle-docking protein FtsY |
| rRNA_mod_*rlmN* | 23S rRNA (adenine(2503)-C(2))-methyltransferase |
| era | GTP-binding protein Era |
| Obg_*cgtA* | Obg family GTPase CgtA |
| L27 | ribosomal protein bL27 |
| TypA_*bipA* | GTP-binding protein TypA/BipA |
| pyrH_bact | UMP kinase |
| *mfd* | transcription-repair coupling factor |
| *rpoC*_TIGR | DNA-directed RNA polymerase, beta' subunit |
| *aspS*_bact | aspartate--tRNA ligase |
| *ksgA* | ribosomal RNA small subunit methyltransferase A |
| *bfr* | bacterioferritin |
| *nrdJ*_Z | ribonucleoside-diphosphate reductase, adenosylcobalamin-dependent |
| cysta_beta | cystathionine beta-synthase |
| guanyl_kin | guanylate kinase |
| TIGR00302 | phosphoribosylformylglycinamidine synthase, purS protein |
| *pta* | phosphate acetyltransferase |
| *purA* | adenylosuccinate synthase |
| *glmM* | phosphoglucosamine mutase |
| TIGR00343 | pyridoxal 5'-phosphate synthase, synthase subunit Pdx1 |
| GTPase_*engA* | ribosome-associated GTPase EngA |
| *pth* | aminoacyl-tRNA hydrolase |
| *trmD* | tRNA (guanine(37)-N(1))-methyltransferase |
| *sufB* | FeS assembly protein SufB |
| *purN* | phosphoribosylglycinamide formyltransferase |
| *recR* | recombination protein RecR |
| *trpB* | tryptophan synthase, beta subunit |
| *rplB*_bact | ribosomal protein uL2 |
| *ffh* | signal recognition particle protein |
| *sdhA*_forward | succinate dehydrogenase, flavoprotein subunit |
| *purF* | amidophosphoribosyltransferase |
| *pheS* | phenylalanine--tRNA ligase, alpha subunit |
| *dapB* | 4-hydroxy-tetrahydrodipicolinate reductase |
| *rpoZ* | DNA-directed RNA polymerase, omega subunit |
| *rplT*_bact | ribosomal protein bL20 |
| *rpsB*_bact | ribosomal protein uS2 |

**
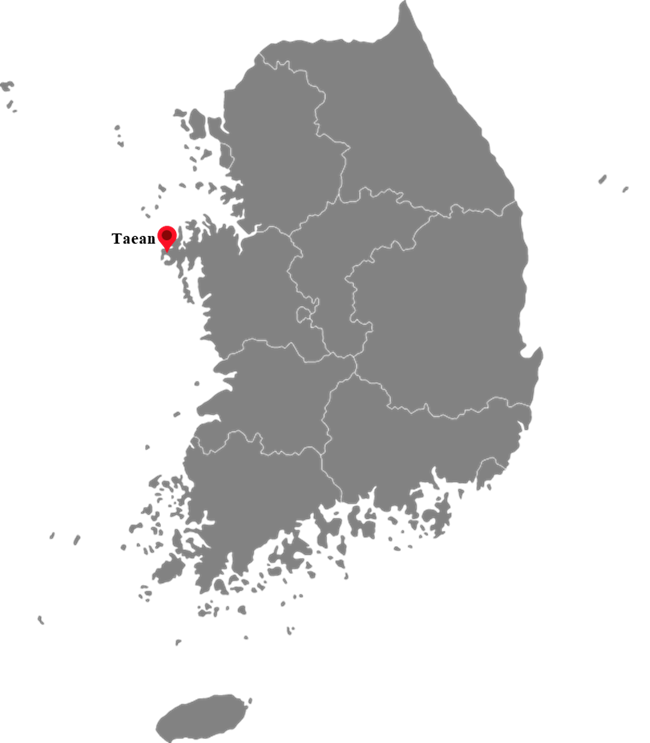
**

**Fig. S1** Location of the sampling sites in South Korea. Red marks indicate areas where soil samples were obtained.

**
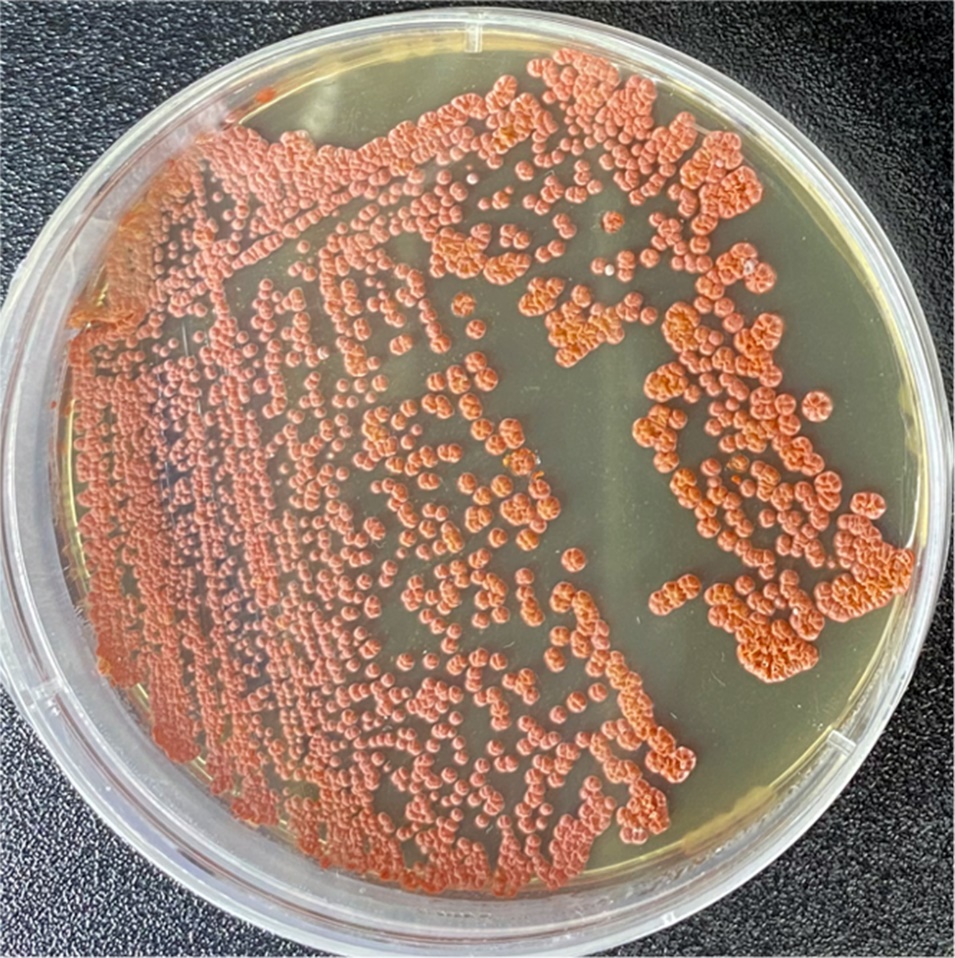
**

**Fig. S2** Morphological characteristics of *Streptomyces spectabilis* KCTC9218^T^ grown on Bennett’s agar media.

**
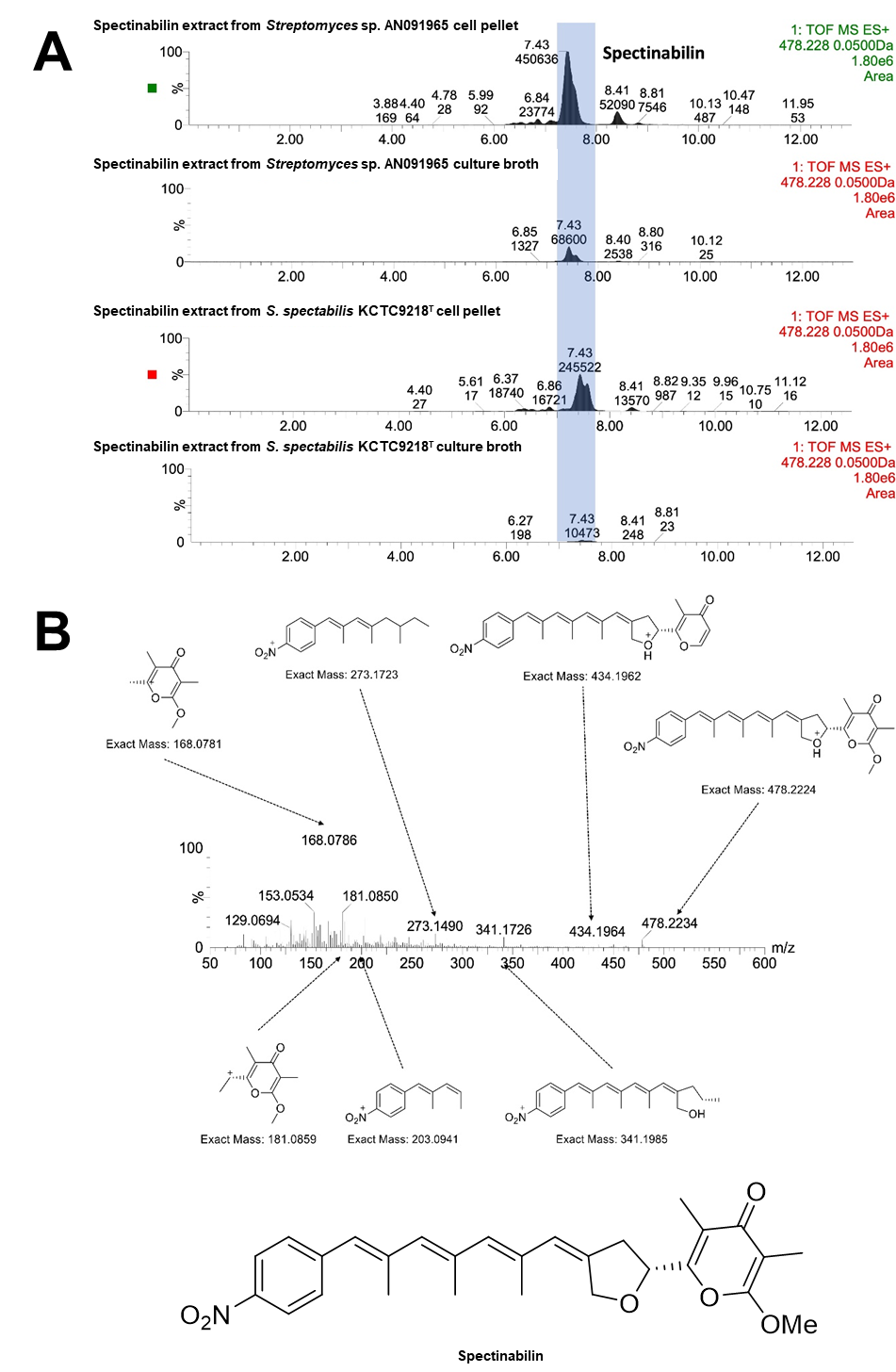
**

**Fig. S3** LC-MS analysis of (A) spectinabilin extracted from the cell pellets and media broth of *S. spectabilis* KCTC9218^T^ and *Streptomyces* sp. AN091965 cultures and (B) their MS/MS fragmentation analysis.


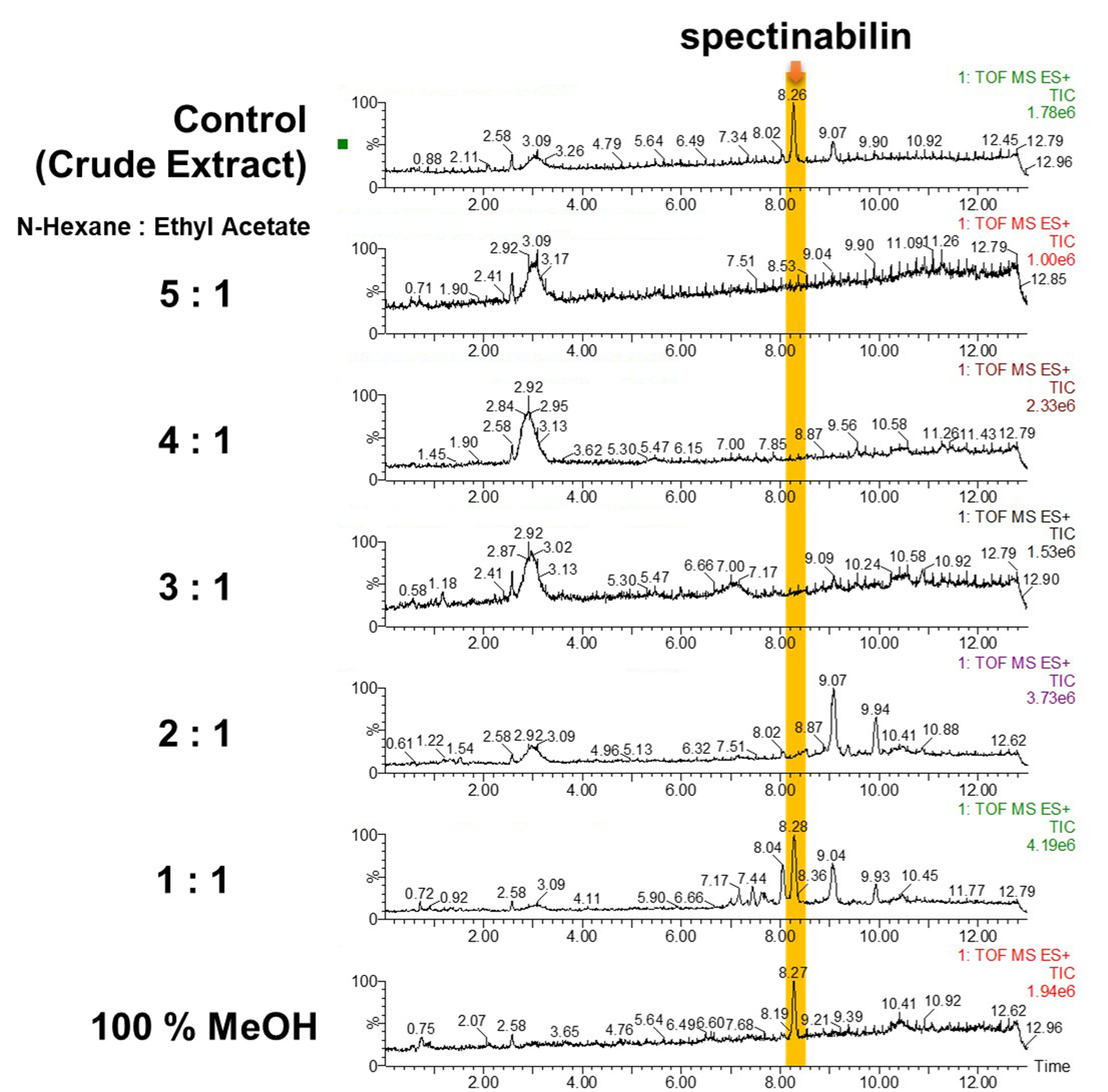


**Fig. S4** LC-MS chromatogram of metabolite fractions collected during purification of spectinabilin from the crude extract of *Streptomyces* sp. AN091965 culture broth and cell pellets. Spectinabilin was eluted from silica column at 100 % methanol.

**
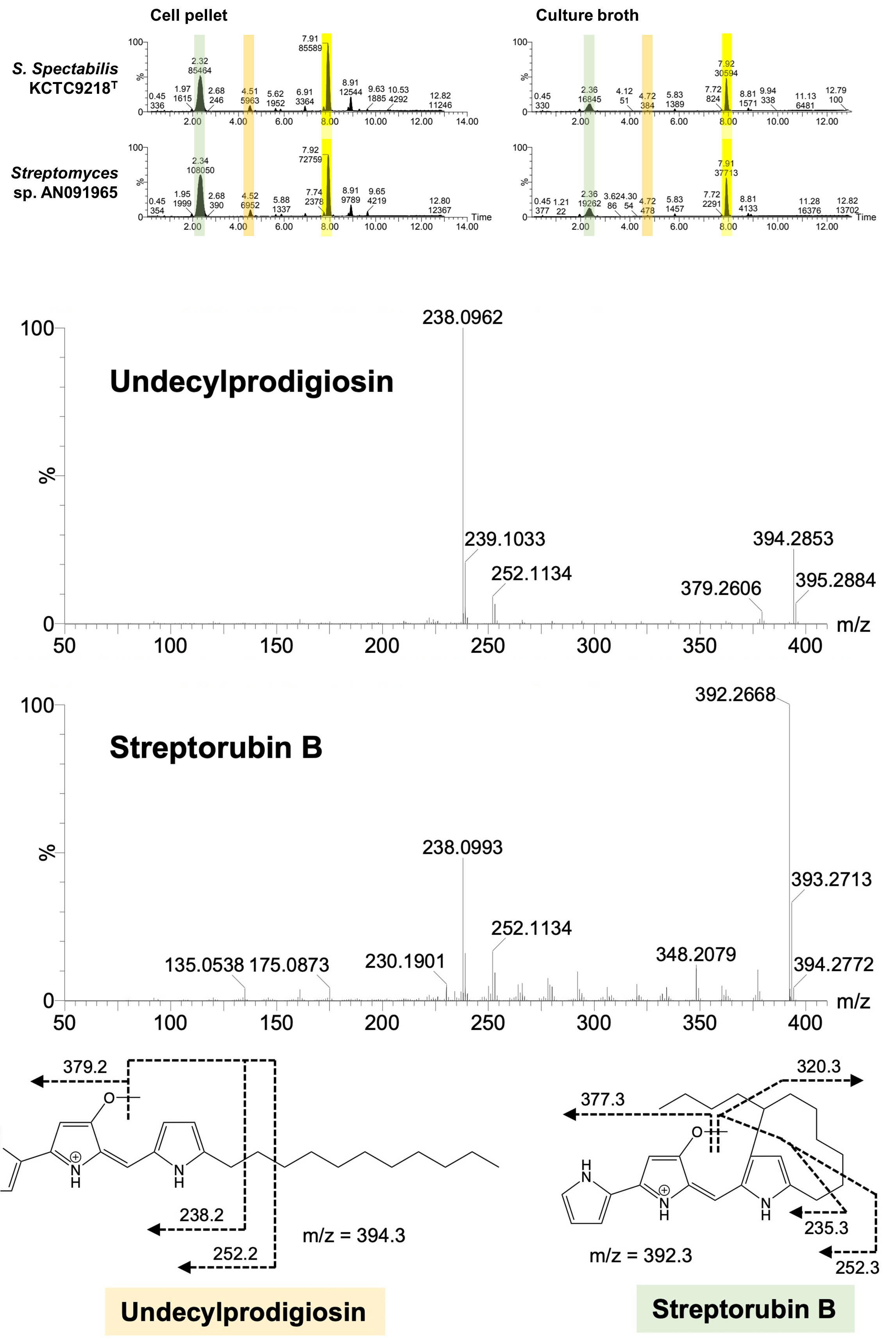
**

**Fig. S5** LC-MS spectrum of spectinabilin (yellow), undecylprodigiosin (orange), and streptorubin B (green) from *S. spectabilis* KCTC9218^T^ and *Streptomyces* sp. AN091965 cultures.
